# To assess and compare the mental health of current-left-behind children, previous-left-behind children with never-left-behind children

**DOI:** 10.3389/fpubh.2022.997716

**Published:** 2022-09-06

**Authors:** Guangyan Yang

**Affiliations:** Department of Psychology, School of Education, Xi'an University, Xi'an, Shaanxi, China

**Keywords:** mental health, left-behind children, overall health, migrants, children

## Abstract

The study's goal was to compare the mental health of children who are now left-behind (current-LBC) to children who have never been left-behind (never-LBC). Recruits were culled from rural Chinese schools. The Strengths and Difficulties Questionnaire (SDQ) and the Parent-Adolescent Communication Scale (PACS) were used to examine participants' migratory status, mental health, and parent-child communication (PACS). A total of 2,000 current-, 500 previous-, and 300 never-LBCs in had complete data readily accessible for research and analysis. A number of mental health issues, such as emotional symptoms, conduct, and hyperactivity issues as well as overall challenges were considerably increased when all confounding factors were taken into account in the analysis. Our findings also showed a substantial link between children's overall issues and their inability to effectively communicate with parents. Children suffer long-term consequences as a result of their parents' frequent moves. The mental health of children is closely linked to the quality of communication between parents and children. Migrant parents' ability to comprehend and communicate with their children is critical to their children's development, according to the findings of this study.

## Introduction

The term “left-behind children” (LBC) refers to children aged <16 years living in rural areas whose parents both leave the area for work or one of the parents leaves the area for work while the other loses guardianship. According to the definition of rural Blissed by the Government of China in 2016, there are a total of 9.02 million rural LBC in China. Most of these children are cared for by grandparents or other relatives and are impoverished. In rural China, immigrant parents have abandoned almost 41 million children under the age of 18 that equates to over one in three children (1). Children who were “left-behind in their rural settlements while one or both of their parents went into cities for employment, and who have not lived with them for over 6 months” (2) are defined as LBC under the age of 18. China had the biggest rural-urban migration in human history as urbanization and industrialization accelerated over the previous 40 years. Two hundred and fifty million people, or 31% of the working population nationwide, are rural-urban migrants (3). Preventing the kids from using public services including healthcare, education, and recreation in the places where they relocate (4). Majority of the migrants work in low-paying occupations and live in subpar neighborhoods, which discourages them from bringing their kids to the places where they relocate (5). The LBC problem was made more acute overall particularly in China by all of these variables. A Chinese survey done by (6) using the Child Behavior Checklist scale in students aged 12–17 years in Yantai City found that the prevalence of emotional and behavioral problems was 10.5%. The study showed the factors that increase the risk of emotional and behavioral problems include poor family relationships, negative life events, learning stress, and living in poor rural areas.

The issues with parent-child separation brought on by sporadic work are not exclusive to China. High levels of LBC are prevalent in several lower and middle status income nations (7–9). Theory of family systems offers a helpful foundation for comprehending how these migrant families' children's mental health. According to the hypothesis, there is a connection between a rise in income of family and a decline in parental care, which has conflicting impacts on children's adjustment (10, 11). For instance, although increased cash through remittances may help children have better nutrition and overall health, parental absence may reduce attention, stimulation, and communication, which may result in the onset of psychological and behavioral issues in children. The impacts of parental migration on LBC have been the subject of a substantial amount of documented study. These researches showed that LBC in rural China have higher difficulties in terms of loneliness, despair, anxiety, and behavioral issues than their peers who live with their parents (11–20). The fluidity and complexity of the family connections, however, are not taken into consideration in the majority of research on LBC's mental health (21). For instance, some parents of LBC could relocate to their hometown after a protracted period of job travel. The discrepancies between the current- and the previous-LBC were only partially covered by previously gathered data (15).

Additionally, the crucial family dynamic of parent-child communication (PACS)—which includes verbal and non-verbal exchanges between parents and children—receives less focus (22). It is stressed that one crucial family aspect influencing children's development is PACS, which serves as a barometer of the quality of the parent-child bond (23). Due to parents' rural-to-urban movement, LBC experience the negative consequences of long-term parental absence, which may have a permanent detrimental impact on the parent-child relationship (24). According to certain research on LBC, the degree of their contact with their parents has a significant impact on both their behavioral and mental health (25). Despite these researches, it is still unknown how PACS affects kids' mental health in relation to various types of parental relocation.

## Aim of the study

The current study aims to learn whether there are any differences in the mental health of children who are currently left-behind (current-LBC), who were left-behind in the past (previous-LBC), and who were never-left-behind (never-LBC), as well as whether there are any differences in the mental health of children who were never-left-behind (never-LBC). It also aims to learn how the effectiveness of PACS affects children's mental health across current-LBC, previous-LBC.

## Subject selection criteria

### Inclusion criteria

1. Two counties with high LBC rates in rural regions of Shaanxi Province were chosen. From each county, two towns were chosen at random, for a total of four towns. The research was subsequently expanded to include two arbitrary schools in each chosen municipality.
2. Students in Grades 7–10 to participate from each school between the ages of 11 and 20 years.
3. Children who were willing to participate in the study.
4. Psychologically healthy students.

### Exclusion criteria

1. To guarantee that participants had the level of literacy required to complete the questionnaire, younger children were excluded from the study.
2. Students, whose parents were deceased, divorced, or remarried.
3. Children who were orphans or raised by a single parent were excluded from the study because the effects on children of various forms of parental absence may vary.
4. Children with any psychological or mental disorder.

## Material and methodology

The present research used self-reported questionnaires and was a cross-sectional survey. Participants were chosen from rural regions in China, a relatively undeveloped province in China's southeast that serves as a hub for migrant workers and has 4.5 million LBC in addition to 16 million migrant workers.

### Ethical clearance and informed consent

Ethical and Research board of Xi'an University provided ethical permission and county authorities local approval was obtained before the start of the research. Furthermore, consent of the individual head teachers was obtained for the research to be carried out at their respective institutions. Parents or guardians of the eligible children and their written informed permission were sought before to the survey.

### Collection of sample

We tried to choose regions with a lot of LBC so that sampling would be simple. For this purpose, we gathered data on the child population from the 2015 National Inter-Census Survey (1).

The student was invited to complete a self-administered questionnaire in their classroom if permission was granted on both forms. The questionnaire was given out in the classroom by the researchers without any teachers or school officials present because of how sensitive some of the questions were. Even after obtaining agreement, participants were informed that there was no need to complete the questionnaire. Confidentiality and anonymity were guaranteed. Measures characteristics of the population.

### The clinical data extraction and analysis

Participant demographics were gathered in the form of their gender, age group, grade level, number of siblings, and family economic position (much better off/better off, the same, or substantially worse). The data was collected by a well-aware and knowledgeable observer and only one observer collected the data to avoid the observer bias.

Students were given two distinct questions: “Did your father (and mother) relocate into cities for work and does he (and she) no longer reside with you for the previous 6 months?” to establish the status of parental migration. The responses were “no, never,” “yes, in the past migrated,” and “yes, in the present moved.” If neither parent moved, the participant was categorized as “never-LBC,” “current-LBC,” or “previous-LBC.” If neither parent migrated, the participant was categorized as “never-LBC,” “current-LBC,” or “previous-LBC”.

### Questionnaires regarding the surveys of strength and difficulties

The psychological and behavioral status of students were evaluated by the Chinese version of Strength and Difficulties Questionnaires (SDQ). The scale contains five dimensions: emotional symptoms, conduct problems, hyperactivity, peer problems, and pro-social behavior. Each dimension has five items, and each item is scored on a three-point Likert scale from 0 (totally non-compliant) to 2 (fully compliant). The total difficulties score is the sum of all dimension scores except the pro-social dimension, and a higher score reflects increased severity of emotional and behavioral problems. The Chinese student version of the SDQ, which has been extensively utilized and validated in the Chinese setting, was used to assess children's mental health (26, 27). The student version of the SDQ is seen to be more appropriate for this age group than the parent or teacher versions (28). Twenty-five mental health-related questions make up the SDQ's five subscales, which include conduct issues, peer issues, emotional symptoms, pro-social behaviors, and hyperactivity. Each item is rated on a Likert scale of 0–2, with 0 signifying uncertainty and 2 denoting absolute certainty. Each subscale's score is calculated by adding together each of its five components. The pro-social subscale was excluded from the calculation of the overall problems score, which ranged from 0 to 40. Higher scores indicate a poorer feeling of well-being and more problematic behaviors across all subscales (with the exception of the pro-social conduct subscale).

### Child-parent communication

The communication between parent and child was assessed with the Chinese version of the Parent-Adolescent Communication Scale (PACS). The scale has 20 items, and includes the open family communication sub-scale (10 items) and the problems in family communication sub-scale (10 items). The open family communication sub-scale measures the free exchange of ideas and feelings between parent and children. The problems in family communication sub-scale measures the willingness of parents and children to honestly express their true thoughts and feelings to each other.

### Five-point Likert scale

Five-point Likert scale measurements are from 1 (strongly disagree) to 5 (strongly agree) in the original version. In order to avoid neutral attitude feedback, we adjusted all items to a four-point Likert scale (1 = strongly disagree, 2 = disagree, 3 = agree, 4 = strongly agree) in the current study. The total PACS score ranges from 20 to 80, and higher scores mean better communication.

Participants score a set of 20 statements on the spectrum from “strongly agree” to “strongly disagree” on a five-point scale. The “open family communication” subscale and the “issues in family communication” subscale are the two subscales of the PACS. Ten items make up the “open family communication” subscale, including “I find it simple to discuss concerns with my father/mother” and “My father/mother attempts to understand my point of view.” Ten items make up the subscale for “issues in family communication,” including “I don't believe I can tell my father/mother how I truly feel about certain things” and “I am sometimes frightened to ask my father/mother for what I want.” The values of the scores for the “problems” subscale items are inverted. Consequently, a high rating suggests that there aren't any communication issues. An additive total scale score is produced by this conversion; higher scores indicate better communication between parents and adolescents. The participants were asked to assess their father and mother's communication individually.

### Connor-Davidson Resilience Scale

The Chinese version of the Connor-Davidson Resilience Scale (CD-RISC) was used to assess children's psychological resilience The English version has 25 questions that measure psychological well-being across five dimensions: control, positive acceptance of change, tenacity, and tolerance of negative affect. These 25 elements were retained in the Chinese version, which also made revisions in the areas of tenacity, strength, and optimism. Each response is evaluated on a Likert scale of 1 (never) to 5 (always) (often). The total of the scores for each sub-items scale's served as the measurement for each dimension. The scores of the three dimensions were added together to get a final score (thus ranging from 25 to 125). Better psychological resiliency is indicated by higher scores. The test-retest reliability (*r* = 0.112, *p* < 0.001) and split-half reliability (*r* = 0.180, *p* < 0.001) of this Chinese version scale were excellent in a research conducted in the south province of China (0.718, *r* = 0.610, *p* < 0.001).

Gender, age, grade, siblings, and self-reported family economic position in comparison to others in their neighborhood (far better off/better off/the same, poorer/much poorer) were all covariates in this research. The majority of recent studies categorize the results of their survey into two groups: children who were left-behind and children who were never-left-behind.

### Analytical statistics

To begin with, chi-square tests or analyses of variance were used to evaluate sample characteristics across the three groups of kids with various parental migration statuses. Second, an ANOVA was used to assess PACS and SDQ differences across groups. Using the criteria for the least significant difference, a *post-hoc* comparison was performed. Finally, to investigate the relationships between parental migration status and mental health outcomes, multiple linear regression models were used. The fundamental model took into account the demography of children (Income status, gender, grade of schools, and presence of siblings). To account for the efficacy of parent-adolescent communication, including both father- and mother-adolescent communication, more modifications were made to the model. The 95% confidence interval and regression coefficients are shown. Additionally studied were the relationships between variables and migration status. SPSS 24.0 (IBM, Armonk, NY, USA) for Windows was used for all statistical analysis.

## Results

### Social and demographic traits

**Figure 1:**
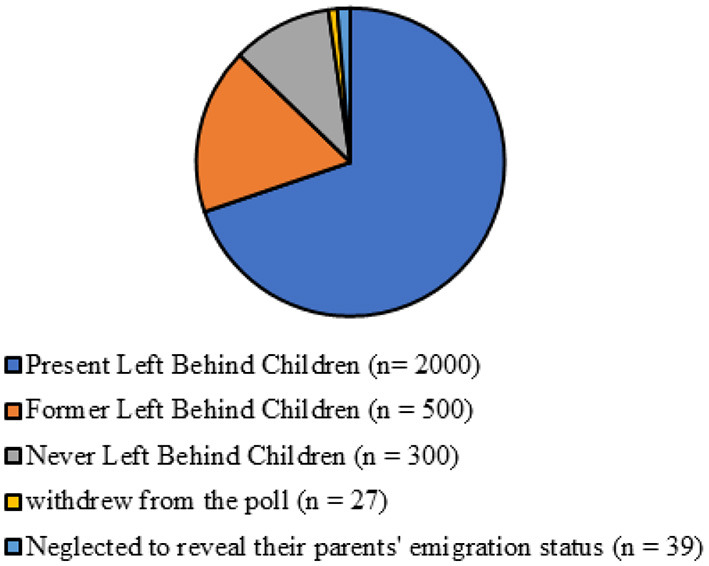
Study participants.

**Figure 2:**
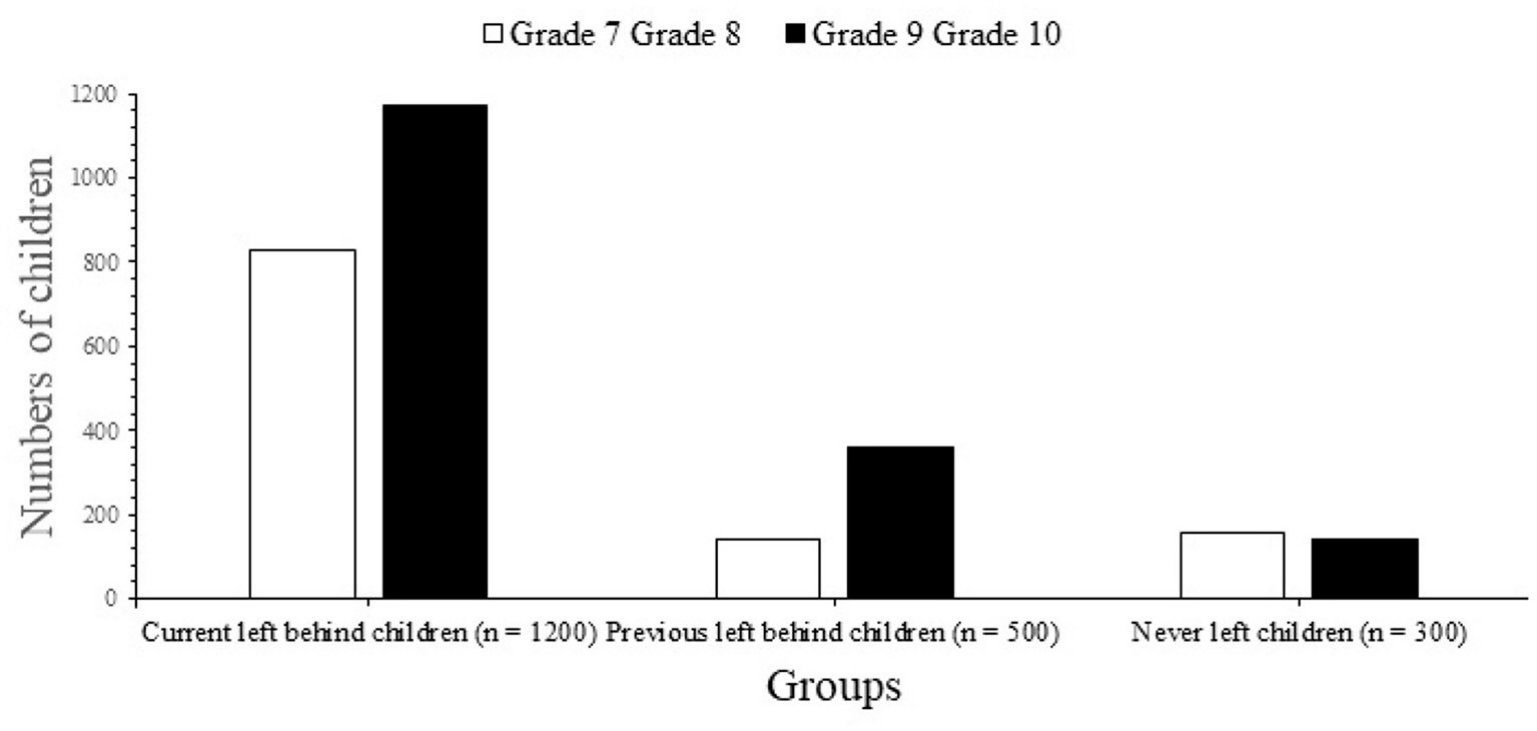
Distribution of grades of school among children.

We received responses from almost 2,800 people: 2,000 present-LBC, 500 former-LBC, and 300 never-LBC. Twenty-seven students (1.3%) withdrew from the poll, while 39 (1.9%) neglected to reveal their parents' emigration status (Figure 1). The participant's demographics are shown in Table 1. According to the overall results, there were 560% more male respondents than female respondents in all three categories. Children in the current-LBC and never-LBC groups were somewhat older on average (mean 13.2 years, SD 1.2 years) than those in the preceding LBC group. About a third of the children were the only ones in each of the three groups. Self-reported socioeconomic status did not vary significantly across groups except grade of schools (Figure 2).

**Table 1:** Sample attributes broken down by parental migration status, *n* (%), left-behind children.

**Variables**	**Current-left-behind children group**	**Previous-left-behind children group**	**Never-left-behind children group**	**Chi square test**	***P*** **-value**
**Gender**				2.08	0.414
Male	1,200 (60.0)	300 (60.0)	180 (60.0)		
Female	800 (40.0)	200 (40.0)	120 (40.0)		
**Age, SD**	14.1 (1.2)	14.2 (1.2)	14.0 (1.2)	5.25	0.011
**Grade**				6.16	0.011
Grade 7, Grade 8	830 (41.5)	140 (28.0)	158 (52.6)		
Grade 9, Grade 10	1,170 (58.5)	360 (72.0)	142 (47.4)		
**Any siblings**				2.69	0.01
Yes	1,400 (70.0)	300 (60.0)	250 (83.3)		
No	600 (30.0)	200 (40.0)	50 (16.7)		

### Parent-Adolescent Communication Scale and the strengths and difficulties questionnaire score comparison

**Figure 3:**
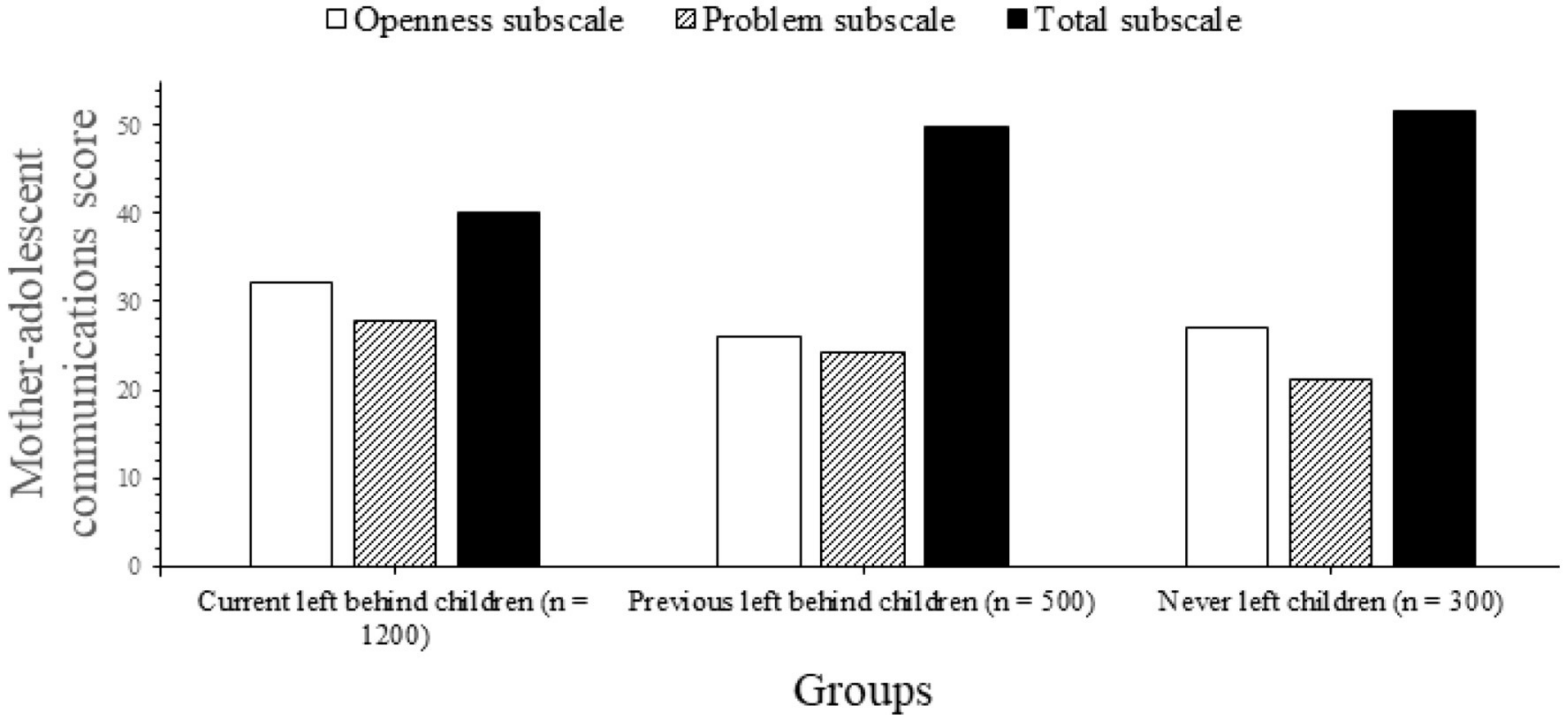
Mother-adolescent communication score.

**Figure 4:**
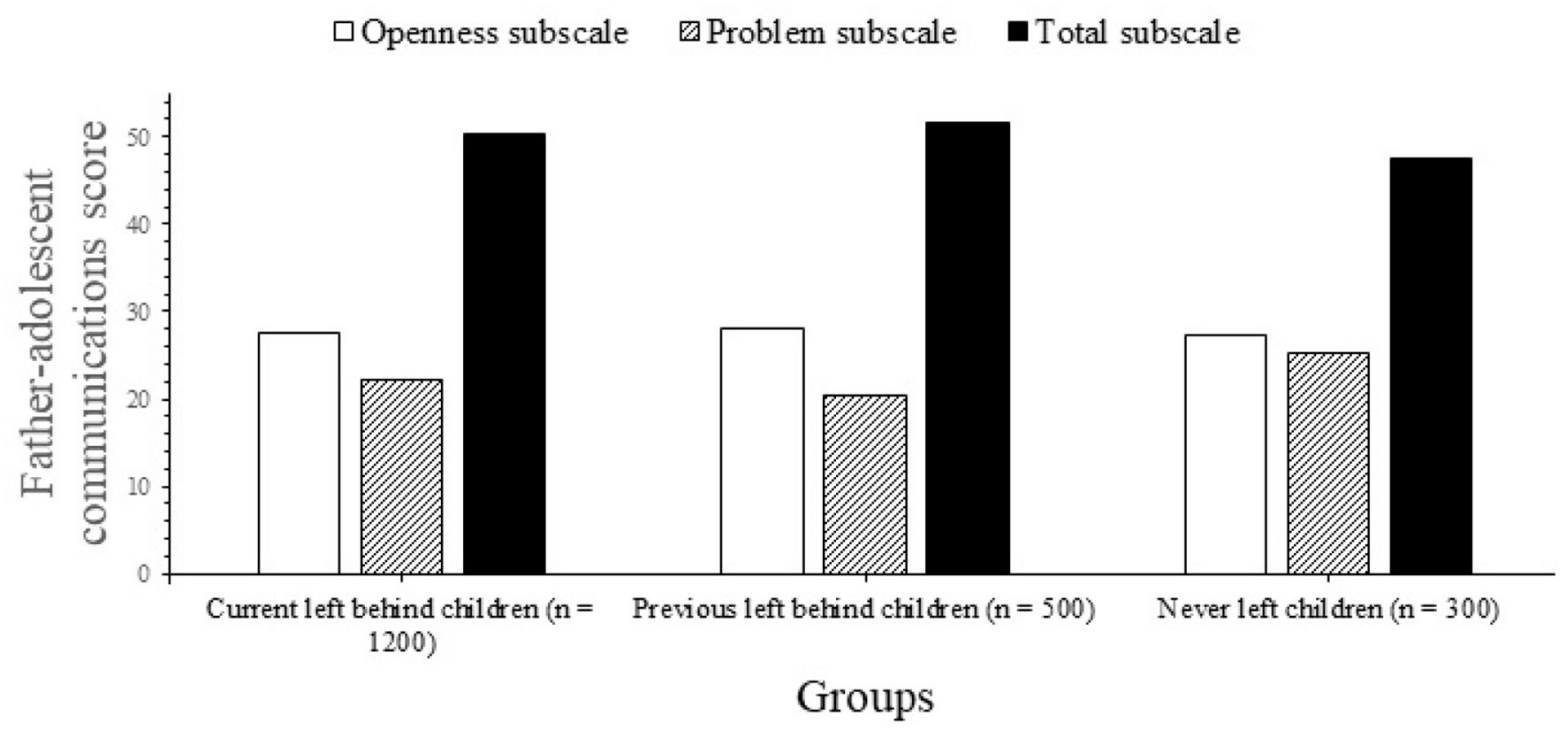
Father-adolescent communication score.

Table 2 summarizes the key results from mean PACS scores across all subscales, including comparisons by age group. There were considerable variations between groups in all PACS categories except for communication issues between dads. There were significant differences in overall assessments, difficulty (with mother), and openness between the previous-LBC group and the never-LBC group for both parents (both with mother and father, Figures 3, 4). *Post-hoc* testing indicated no significant variations in total or subscale scores between the current-LBC and previous-LBC, with the exception of the overall communication with mothers score. For each subscale and overall difficulty level, the average SDQ results for the three kid groups are shown in Table 2. All three difficulty subscales, as well as the overall difficulty score, were considerably greater for current- and previous-LBC (emotional signs, conduct issues, and status of hyperactivity). According to *post-hoc* assessments, there were no significant changes in total or subscale scores between current- and former-left-behind students.

**Table 2:** Comparison of the results of the Parent-Adolescent Communication Scale (PACS) and the Strengths and Difficulties Questionnaire (SDQ) for those who have had LBC in the past, are now LBC, and have never had LBC (SD).

**Variables**	**Current-LBC**	**Previous-LBC**	**Never-LBC**		***F*** **_PC_**
**Mother-adolescent communication**					
Openness subscale	32.1	26.1	27.1	3.68^*^	(2,3)
Problem subscale	27.7	24.2	21.2	3.33^*^	(2,3)
Total scale	40.2	49.7	51.7	4.12^*^	(1,2) (2,3)
**Father-adolescent communication**					
Openness subscale	27.6	28.1	27.2	4.16^*^	(2,3)
Problem subscale	22.2	20.3	25.2	2.29	
Total scale	50.2	51.6	47.6	5.00^**^	(2,3)
**SDQ**					
Emotional symptoms	3.7	3.1	3.5	7.21^**^	(1,3) (2,3)
Conduct problems	2.1	2.9	2.4	4.78^**^	(1,3) (2,3)
Hyperactivity	4.9	4.9	3.3	8.12^***^	(1,3) (2,3)
Peer problems	2.3	2.1	2.2	1.11	
Total difficulties score	12.6	12.2	11.2	9.02^***^	(1,3) (2,3)
Pro-social	6.1	7.1	7.9	1.00	

* *p* < 0.05,

** *p* < 0.01,

*** *p* < 0.001.

### Association with SDQ subscale scores

**Figure 5:**
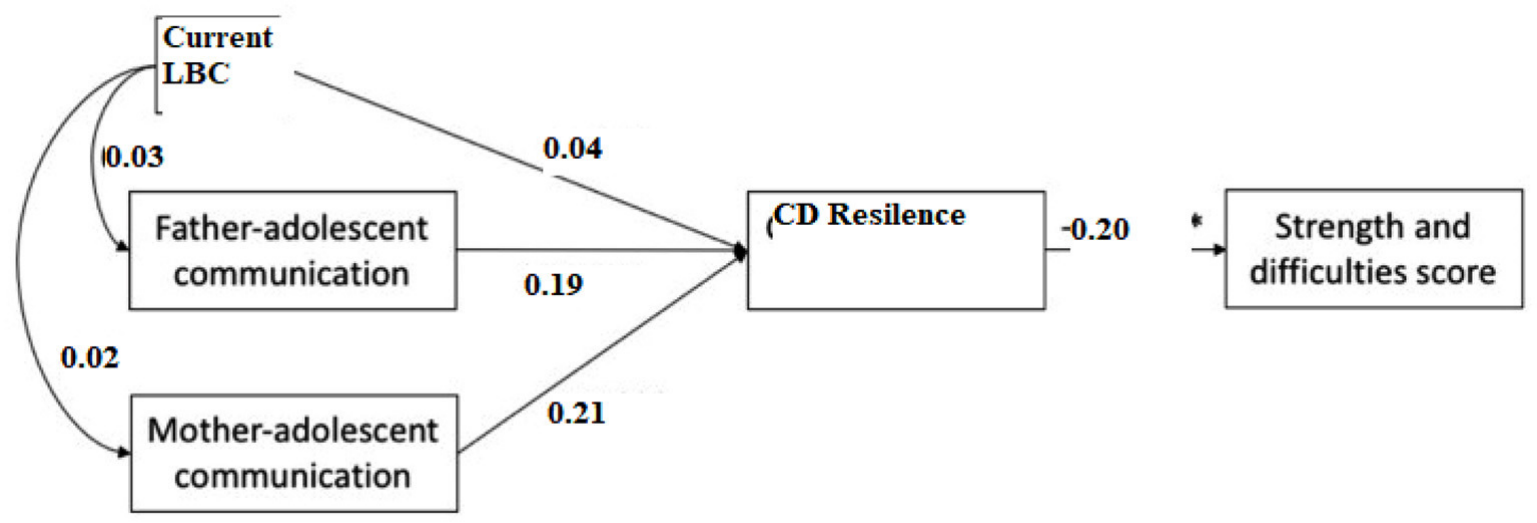
Flow diagram.

Table 3 shows the regression results for the SDQ subscale scores. Behavioral problems and hyperactivity were shown to be significantly associated with parental absence, even after accounting for all potential confounders. Emotional symptoms in the current-LBC group were more severe than in the previous-LBC group when compared to never-LBC. Girls outperformed boys when it came to emotional issues. Higher grades (Grades 9 and 10) were shown to have higher levels of hyperactivity but less behavioral difficulties compared to younger students (Grades 7 and 8). Emotional symptoms, behavioral problems, and hyperactivity were all linked to poor communication with your parents. They were less likely to develop hyperactivity issues if they had more open communication with their parents (mother or father) (Figure 5).

**Table 3:** The SDQ's regression analysis by kind of kid, parent-adolescent communication, and demographic characteristic (emotional disorders, behavioral difficulties, hyperactivity).

**Variables**	**Sign of emotions^#^**	**Conduct issues^$^**	**Status of hyperactivity^&^**
**Migration status of parents (ref never-LBC)**			
Current-left-behind children	0.40 (0.21, 0.47)^***^	0.32 (0.01, 0.42)^**^	0.63 (0.12, 0.70)^***^
Previously-left-behind children	0.31 (−0.01, 0.51)	0.29 (0.02, 0.19)^*^	0.23 (0.01, 0.53)^*^
**Gender (ref male)**			
Female	0.59 (0.36, 0.53)^***^	−0.04 (−0.11, 0.04)	0.20 (−0.04, 0.18)
**Grade of children in schools** (**ref Grade 7, Grade 8)**			
Grade 9, Grade 10	−0.21 (−0.23, 0.02)	−0.21 (−0.20, 0.01)^*^	0.21 (0.12, 0.34)^***^
**Level of income (ref: much better)**			
Poorer/much poorer	0.28 (−0.05, 0.61)	0.15 (−0.11, 0.40)	0.11 (−0.21, 0.43)
**Siblings (reference: yes)**
No	−0.14 (−0.34, 0.05)	−0.08 (−0.23, 0.07)	−0.20 (−0.38, −0.01)^*^
**Communication regarding mother-adolescent**			
Scale of openness	0.02 (0.04, 0.01)	0.01 (0.01, 0.01)	0.04 (0.05, 0.01)^***^
Scale of problem	0.10 (0.07, 0.11)^***^	0.05 (0.02, 0.12)^***^	0.07 (0.07, 0.11)^***^
**Communication regarding father-adolescent**			
Scale of openness	0.01 (0.03, 0.01)	0.01 (0.01, 0.02)	0.03 (0.05, 0.01)^*^
Scale of problem	0.04 (0.01, 0.06)^**^	0.04 (0.02, 0.06)^**^	0.04 (0.01, 0.07)^**^

* *p* < 0.05,

** *p* < 0.01,

*** *p* < 0.001,

# adjusted *R*^2^ = 0.163,

$ adjusted *R*^2^ = 0.120,

& adjusted *R*^2^ = 0.201.

### Relationship to SDQ total difficulty scores

Table 4 shows the correlation between parental migration status and overall SDQ issues. Both the current- and prior-LBC were much more likely to have higher total difficulty scores. After adjusting for all confounding variables, the total issues score increased significantly in current-LBC (*p* < 0.001) and previous-LBC (*p* < 0.05) compared to never-LBC. In the baseline model (0.01, *p* < 0.05), older students (Grades 7 and 8) and those who had siblings were the only ones shown to be more vulnerable. When all other characteristics were taken into account, children from low-income families were more likely to have more overall difficulties (*p* < 0.05). There were positive associations between difficulties and communication concerns with parents. Furthermore, free communication with one's mother rather than one's father was linked to greater happiness (*p* < 0.001).

**Table 4:** The total challenges related to parental migration status with and without communication adjustment for parents and adolescents.

**Variables**	**Baseline^#^**	**Adjusted^$^**
**Migration status of parents (ref: never-LBC)**		
Current-left-behind children	1.41 (0.50,1.22)^***^	1.45 (0.60, 1.20)^***^
Previously-left-behind children	1.30 (0.49,2.22)^**^	0.50 (0.04, 1.45)^*^
**Gender (reference: male)**		
Female	0.11 (−0.23, 0.47)	0.32 (−0.05, 0.71)
**Grade of children in schools** (**ref Grade 7, Grade 8)**		
Grade 7, Grade 8	0.89 (0.29, 1.22)^**^	−0.23 (−0.77, 0.01)
**Level of income (ref: much better)**		
Poorer	1.97 (0.60, 1.31)^***^	1.44 (0.15, 1.30)^*^
**Siblings (reference: yes)**		
No	−0.52 (−1.09, −0.03)^*^	−0.31 (−0.34, 0.04)
**Communication regarding mother-adolescent**		
Scale of openness		0.21 (0.12, 0.03)^***^
Scale of problem		0.29 (0.21, 0.31)^***^
**Communication regarding father-adolescent**		
Scale of openness		0.02 (0.10, 0.01)
Scale of problem		0.12 (0.07, 0.28)^***^

* *p* < 0.05,

** *p* < 0.01,

*** *p* < 0.001,

# adjusted *R*^2^ = 0.022,

$ adjusted *R*^2^ = 0.244.

The SEM findings revealed that the fit for each of the three models was adequate. All of comparative fit index (CFI) and Tucker-Lewis index > 0.81, and root mean square error of approximation <0.062 (Table 5).

**Table 5:** Multigroup model.

	**Chi-square test**	***df***	**Comparative fit index**	**Tucker-Lewis index**	**Root mean square error of approximation**
Current-LBC	180.111	10	0.899	0.967	0.078
Previously-LBC	172.564	10	0.999	1.001	0.002
Never-LBC	168.912	10	0.892	0.987	0.065

## Discussion

In order to examine the impact of parental relocation on children's mental health, the current study aimed to compare current-LBC with previous-LBC. Researchers have documented several aspects of child well-being and how they are linked to family dynamics and family relationships in their comprehensive study. We found that migrant children are much more likely than non-migrant children to report higher levels of emotional symptoms, higher levels of behavioral difficulties, and higher levels of hyperactivity. This featured both present- and former-children of the LBC. Interaction between parents and children has been shown to have a positive effect on children's mental health. Our findings shed light on a slew of pressing issues and provided information that might be used to the development of effective intervention strategies.

First and foremost, our findings show that children's mental health has been negatively impacted by both their previous- and current-experience of long-term separation from their migrant parents, and these effects are both large and long lasting. After adjusting for the most important confounding variables, this was the result. Parents who relocate with their children have a long-term impact on their children's emotional and behavioral issues, which is consistent with previous studies (29, 30). Migrant parents returning to their children's life may not be able to repair the impacts of their long absence. This may be the case for a number of different reasons. Since migration denotes a change in primary caregiver (31), the return of a parent may provide new challenges for their child. In a previous qualitative study in rural Zhejiang Province, 17 migrant parents were interviewed in detail, and the findings showed that some migrants chose to return home because of their child's growth or the occurrence of some unpleasant situations. Left-behind children's hazards must be taken into consideration in any future study. Second, poor parent-adolescent communication has a significant impact on children's mental health. Parents' migratory status and communication with their teenage children did not seem to have any significant interaction effects, however. Attachment theory (32) provides a framework for children in families where communication between parents and adolescents is less efficient. Early contacts with parents help children form secure bonds in their relationships with others. It's possible for children who have had their parental contact cut off to develop unstable attachments. According to the attachment theory, children who have insecure attachments are more likely to have internalizing (such as emotional symptoms) and externalizing (such as behavioral difficulties and hyperactivity) problems when they are exposed to stressful situations. As a result, migrant parents should put improving their children's education as a top priority (33–35).

When they're working remotely, they need to be able to communicate effectively and aid their children. Interventions that focus on parent-adolescent communication should be implemented in locations that serve as a launching pad for migrant workers (36–39).

Thirdly, we discovered some interesting demographic tendencies. Intriguingly, the associations between gender, age, wealth, and the presence or absence of siblings and mental health outcomes varied across different measures and even showed contradictory directions on occasion. Additional research is needed to better understand the mechanics of these kinds of connections. Programs for children from varied socioeconomic backgrounds should be developed.

There are several limitations to keep in mind. Firstly, the use of a cross-sectional design restricts these findings. These issues demand more study, which will necessitate a longitudinal approach. Second, extrapolating results from two counties in a single poor Chinese province should be done with caution. A self-reported mental health continuum rather than a professional diagnosis is the third limitation of this study's findings. Care should be used when extending the current results to clinical situations. This study solely focused on a limited number of characteristics, and related subjects such as the children's interactions with their caregivers (such as grandparents, for example) were not included (i.e., family social capital, etc.).

The study's findings, despite these limitations, strongly suggest that parental migration is damaging to LBC's mental health, independent of household circumstances or contact between parents and adolescents. We also emphasize the long-term harmful impacts of being left-behind, which were largely disregarded in previous research. In view of these results, we are encouraged by the discovery of a decrease in relative LBC levels. The number of LBCs dropped from 61 million in 2010 to 41 million in 2015 (1). As part of China's efforts to better protect and care for LBC, this is a positive development. The accumulating evidence of the negative impacts of parental migration on children cannot be ignored, even if banning parents from relocating for work is plainly undesirable and impossible. We must find a way to assist the large number of children who suffer from internalizing and externalizing problems. In light of the strong correlation between parent-adolescent communication and child mental health, local governments, and migrant-sending communities should work to train migrant parents to increase their understanding of the importance of PACS and to concentrate on improving their communication skills with their LBC. Local communities should also do more to aid the most vulnerable members in their community.

This study demonstrated that PACS has almost the same effect on psychological resilience under different parental migration status, and this conforms with previous research on this subject. Communication between parents and children is usually considered as an important factor, and similar findings have also been reported in other studies. Van et al. (40) reported that PACS was a promising factor to focus on in interventions aimed at preventing mental illness, and Elgar et al. (41) reported that PACS during family dinners had 13–30% positive effect on mental health.

## Conclusions

Left-behind children will continue to be widely practiced in China for the foreseeable future, as will the negative effects of parental mobility on children. Through an analysis of the differences between children who are now left-behind and children who were previously left-behind, this study added to the body of knowledge on previous-LBC, who are often disregarded in academic research. Our study contributes to the corpus of knowledge and will have a big impact on how treatments to improve the mental health of LBC in rural China are developed. When children are left-behind, migrant parents should be encouraged to communicate with them on a regular basis and in a positive manner.

## Limitations of the study

Among the limitations of this study was the method of sampling, which was slightly biased due to the self-reporting; therefore, the results may not be generalizable. The student questionnaire was a self-report assessment, and they may not have admitted some of their emotions and behaviors. Moreover, the study was conducted in the presence of the teachers of the schools due to which children might not have expressed their emotions properly. Because of the constraints of the conditions, we were not able to obtain more emotional and behavioral information from the left back children.

## Future indications of the study

This manuscript highlights that psychological resilience is the key-mediating factor associated with parental migration status and PACS. Better psychological resilience is related to fewer psychological problems among different parental migration statuses. To promote the health of LBC, interventions are needed to enhance psychological resilience, such as implementing positive psychology education in school, fostering their communication skill with parents, enhancing cultural adaptation self-efficacy, which may prevent psychological and related behavioral problems. Furthermore, awareness of LBC must be provided to the parents as well as adolescents and studies are required to be conducted from time to time to keep a check over the mental levels of the left back children. Social authorities and public health organizations should be aware that children left-behind are at increased risk of mental health problems. There is an urgent need for the development of better preventive measures and more effective management strategies.

## Data availability statement

The original contributions presented in the study are included in the article/supplementary material, further inquiries can be directed to the corresponding author/s.

## Ethics statement

The studies involving human participants were reviewed and approved by Ethical and Research Board of Xi'an University and county authorities provided local approval. Written informed consent to participate in this study was provided by the participants' legal guardian/next of kin. Written informed consent was obtained from the minor(s)' legal guardian/next of kin for the publication of any potentially identifiable images or data included in this article.

## Author contributions

GY contributed to the aim and objective of the study, data collection, data assessment, writing, and submission.

## Funding

The study was supported by A study on the characteristics and causes of aggressive Development of Migrant Children in Shaanxi, Xi 'an Scientific Planning Fund Office, Shaanxi, China (Grant No. 13WL09).

## Conflict of interest

The author declares that the research was conducted in the absence of any commercial or financial relationships that could be construed as a potential conflict of interest.

## Publisher's note

All claims expressed in this article are solely those of the authors and do not necessarily represent those of their affiliated organizations, or those of the publisher, the editors and the reviewers. Any product that may be evaluated in this article, or claim that may be made by its manufacturer, is not guaranteed or endorsed by the publisher.
